# A focus on copper depletion-induced cuproptosis for cancer therapy

**DOI:** 10.1039/d5sc90034d

**Published:** 2025-02-18

**Authors:** Hongjie Zhang

**Affiliations:** a State Key Laboratory of Rare Earth Resource Utilization, Changchun Institute of Applied Chemistry, Chinese Academy of Sciences Jilin Changchun 130022 China hongjie@ciac.ac.cn; b Department of Chemistry, Tsinghua University Beijing 100084 China

## Abstract

Copper has emerged as a promising target for cancer therapy, with extensive studies on copper accumulation-induced cuproptosis. However, the potential of copper depletion-induced cuproptosis remains largely unexplored. Recently, Zhou *et al.* (M. Zhou, F. Muhammad, Y. Zhang, T. Li, J. Feng, J. Zhao and H. Wei, *Chem. Sci.*, 2025, https://doi.org/10.1039/D4SC04712E) reported an innovative strategy for copper depletion-based cuproptosis. Notably, this approach leverages the solubility product principle, a mechanism not previously addressed in studies, to achieve effective tumor therapy through the disruption of copper homeostasis.

## Abstract 2

Cuproptosis occurs *via* copper overaccumulation and depletion. While overaccumulation is well studied, depletion is less so. Here, copper depletion-induced cuproptosis is examined (Zhou *et al.*, *Chem. Sci.*, 2025, https://doi.org/10.1039/D4SC04712E).

## Graphical abstract

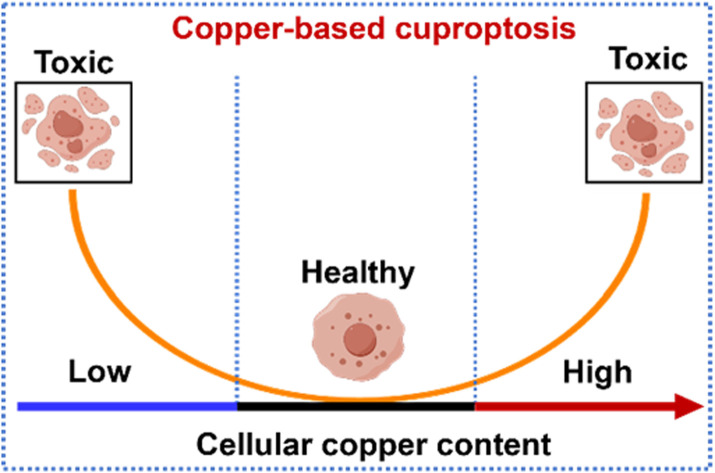

Copper, an essential trace element, is crucial for various cellular processes, including embryonic development, erythrocyte formation, mitochondrial respiration, intracellular redox balance, and biosynthesis of neurotransmitters as cofactors for cellular enzymes.^1,2^ However, copper imbalance can cause diseases such as Menkes disease (caused by copper deficiency), Wilson’s disease (caused by copper accumulation), and is even associated with cancer.^3,4^ In particular, tumor cells typically need a higher copper content to maintain mitochondrial respiration, redox homeostasis, and kinase signaling.^5–8^ At the same time, they must strictly regulate copper content to prevent copper accumulation-induced cell death. Consequently, targeting cellular copper has emerged as a novel strategy for cancer therapy, focusing either on copper depletion or accumulation.^2,9^

The team of Zhou *et al.* has developed an innovative strategy for copper depletion-induced cuproptosis (https://doi.org/10.1039/D4SC04712E).^10^ Copper depletion in this approach is based on the solubility product principle, a concept not previously explored. They chose ZnS nanoparticles as a copper chelator, leveraging a cation exchange reaction between Zn^2+^ and Cu^2+^. This cation exchange reaction is driven by the large difference in solubility product constants (*K*_sp_) between ZnS and CuS. Moreover, they modified ZnS with a core–shell structure to facilitate normal tissue protection and functionalization. Using a tumor-bearing mouse model, they demonstrated inhibition of both primary and metastatic tumors *via* copper depletion-promoted tumor therapy. Additionally, the mechanism of copper depletion was proposed, which was primarily associated with the dysfunction of cellular copper-containing enzymes, in contrast to the aggregation mechanism of copper accumulation-induced cuproptosis (Fig. 1).^11^

**Fig. 1:**
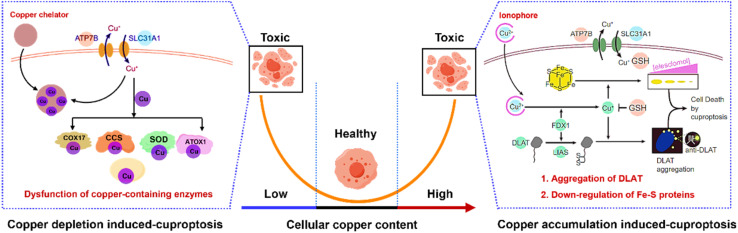
Schematic description of copper-based cell death. (Left) Copper depletion induced-cuproptosis.^10^ (Right) Copper accumulation induced-cuproptosis.^11^

The mechanism of copper accumulation-induced cuproptosis was elaborated on in 2022. It was revealed that excessively accumulated copper ions bind to the lipoylated proteins, such as dihydrolipoamide *S*-acetyltransferase (DLAT) in the tricarboxylic acid (TCA) cycle of the mitochondrial respiratory chain, leading to the aggregation of lipoylated proteins, down-regulation of iron–sulfur cluster proteins, and eventual cell death.^11^ However, copper accumulation-based cuproptosis requires the supplementation of copper ions, which inevitably cause toxic side effects to normal tissues or cells. Notably, the current copper depletion strategy provides an opposite but complementary cuproptosis for effective tumor therapy.

While copper-based molecular drugs are in preclinical or clinical phases,^6,12–16^ their application in tumor therapy remains ambiguous. This study creatively designed a new strategy for copper depletion-induced cuproptosis utilizing nanotechnology, which circumvents the limitation of molecular drugs. However, further studies should be followed to clarify the precise mechanism of copper depletion and to enhance copper-based tumor therapy.

## Author contributions

Hongjie Zhang: writing the commentary, and funding acquisition.

## Conflicts of interest

There are no conflicts to declare.
